# Effectiveness and Timing of Vaccination during School Measles Outbreak

**DOI:** 10.3201/eid1809.111578

**Published:** 2012-09

**Authors:** Axel Antonio Bonačić Marinović, Corien Swaan, Ole Wichmann, Jim van Steenbergen, Mirjam Kretzschmar

**Affiliations:** National Institute for Public Health and the Environment (RIVM), Bilthoven, the Netherlands (A.A. Bonačić Marinović, C. Swaan, J. van Steenbergen, M. Kretzschmar);; University Medical Centre Utrecht, Utrecht, the Netherlands (A.A. Bonačić Marinović, M. Kretzschmar);; Robert Koch Institute, Berlin, Germany (O. Wichmann);; and Leiden University Medical Centre, Leiden, the Netherlands (J. van Steenbergen)

**Keywords:** measles, vaccination, outbreak response, schools, stochastic model, intervention, impact, viruses

## Abstract

Implementing a vaccination campaign during an outbreak can effectively reduce the outbreak size.

## Medscape CME Activity

Medscape, LLC is pleased to provide online continuing medical education (CME) for this journal article, allowing clinicians the opportunity to earn CME credit.

This activity has been planned and implemented in accordance with the Essential Areas and policies of the Accreditation Council for Continuing Medical Education through the joint sponsorship of Medscape, LLC and Emerging Infectious Diseases. Medscape, LLC is accredited by the ACCME to provide continuing medical education for physicians.

Medscape, LLC designates this Journal-based CME activity for a maximum of 1 *AMA PRA Category 1 Credit(s)*^TM^. Physicians should claim only the credit commensurate with the extent of their participation in the activity.

All other clinicians completing this activity will be issued a certificate of participation. To participate in this journal CME activity: (1) review the learning objectives and author disclosures; (2) study the education content; (3) take the post-test with a 70% minimum passing score and complete the evaluation at www.medscape.org/journal/eid; (4) view/print certificate.

**Release date: August 16, 2012; Expiration date: August 16, 2013**

## Learning Objectives

Upon completion of this activity, participants will be able to:

- Assess measles vaccination and the prognosis of measles infection
- Distinguish the maximum amount of time permissible to initiate an outbreak response vaccination in order to prevent a large outbreak of measles
- Distinguish the maximum amount of time permissible to initiate an outbreak response vaccination in order to significantly affect the rate of measles infection at all
- Analyze the peak incidence rates of measles infection during a school outbreak

## CME Editor

**P. Lynne Stockton, VMD, MS, ELS(D),** Technical Writer/Editor, *Emerging Infectious Diseases. Disclosure: P. Lynne Stockton, VMD, MS, ELS(D), has disclosed no relevant financial relationships.*

## CME Author

**Charles P. Vega, MD,** Health Sciences Clinical Professor; Residency Director, Department of Family Medicine, University of California, Irvine. *Disclosure: Charles P. Vega, MD, has disclosed no relevant financial relationships.*

## Authors

*Disclosures: Axel Antonio Bonačić-Marinović, PhD; Corien Swaan, MD; Ole Wichmann, MD; Jim van Steenbergen, MD, PhD; and Mirjam Kretzschmar, PhD, have disclosed no relevant financial relationships.*
