# Supplementary material for: Effectiveness and Timing of Vaccination during School Measles Outbreak
Source: Emerg Infect Dis. 2012 Sep;18(9):1405–13. doi: 10.3201/eid1809.111578 (PMC3437694; doi:10.3201/eid1809.111578)
Supplement: Related texts

# Research article: Effectiveness and Timing of Vaccination during School Measles Outbreak

**DOI:** 10.3201/eid1809.111578

**Authors:** Axel Antonio Bonačić Marinović, Corien Swaan, Ole Wichmann, Jim van Steenbergen, Mirjam Kretzschmar

**Affiliations:** National Institute for Public Health and the Environment (RIVM), Bilthoven, the Netherlands (A.A. Bonačić Marinović, C. Swaan, J. van Steenbergen, M. Kretzschmar);; University Medical Centre Utrecht, Utrecht, the Netherlands (A.A. Bonačić Marinović, M. Kretzschmar);; Robert Koch Institute, Berlin, Germany (O. Wichmann);; and Leiden University Medical Centre, Leiden, the Netherlands (J. van Steenbergen)

## Abstract

Despite high vaccination coverage in most European countries, large community outbreaks of measles do occur, normally clustered around schools and resulting from suboptimal vaccination coverage. To determine whether or when it is worth implementing outbreak-response vaccination campaigns in schools, we used stochastic outbreak models to reproduce a public school outbreak in Germany, where no vaccination campaign was implemented. We assumed 2 scenarios covering the baseline vaccination ratio range (91.3%–94.3%) estimated for that school and computed outbreaks assuming various vaccination delays. In one scenario, reacting (i.e., implementing outbreak-response vaccination campaigns) within 12–24 days avoided large outbreaks and reacting within 50 days reduced outbreak size. In the other scenario, reacting within 6–14 days avoided large outbreaks and reacting within 40 days reduced the outbreak size. These are realistic time frames for implementing school outbreak response vaccination campaigns. High baseline vaccination ratios extended the time needed for effective response.

Measles is a highly contagious disease that causes illness and death in developing and industrialized countries. Measles has an estimated basic reproduction number (R_0_) range of 12–40 (*1*–*5*), meaning that 1 case introduced into a susceptible (naive) population will produce, on average, that number of secondary cases. Worldwide, measles is the main vaccine-preventable cause of death among children; >31 million cases occur every year, and case-fatality rates for industrialized countries are ≈0.1%–0.2% (*6*). In 2001, the World Health Organization (WHO) and the United Nations published a global strategic plan for measles (*7*); the aims of the plan are to sustainably reduce deaths from measles and to interrupt measles virus transmission in countries and regions with elimination objectives.

In 2002, the WHO Region of the Americas was declared free from endemic measles transmission, which was achieved by implementing immunization programs with very high vaccination coverage (>95%). This goal has not been achieved in the WHO European region, for which the target year for measles elimination was 2010. Measles is so highly contagious that the average vaccination coverage in Europe (80%–95%) (*8*) is not high enough to prevent outbreaks among nonvaccinated persons. The new target year for measles elimination in the WHO European region is 2015 (*9*).

The only means of protection against measles are prior infection or vaccination. Several studies have focused on the effectiveness of mass outbreak-response vaccination campaigns for controlling measles outbreaks in settings where incidence and morbidity and mortality rates are high. Although some studies suggest that mass outbreak-response vaccination campaigns will not stop measles epidemics because of the rapid spread of the disease (*10*–*12*), other studies, which used more recent data, show that outbreak-response vaccination campaigns can successfully reduce illness and death (*13*–*17*). The current WHO guidelines recommend mass outbreak-response vaccination campaigns when a measles outbreak is confirmed in settings with a goal of reducing deaths from measles (*18*) and where most measles cases occur in children <5 years of age. There is no recommendation, however, for implementing outbreak-response vaccination campaigns in settings where incidence and morbidity and mortality rates are low, such as in the WHO European region. In these settings, because of the high baseline vaccination ratio (BVR), a shift in age distribution of measles cases toward older nonvaccinated school children is often noted (*19*). Consequently, outbreaks initially spread within relatively closed populations of children, such as schools or daycare centers, because of the high rate of social contact among nonvaccinated children in these establishments. Therefore, we focused our study on outbreak-response vaccination campaigns that targeted establishments with children where a measles outbreak was occurring in settings with high BVRs.

A delay from detection of an outbreak to implementation of an outbreak-response vaccination campaign to onset of an effective immune response of those vaccinated is inevitable. Because measles is highly contagious, many children might become infected during that delay. Thus, whether vaccinating children against measles during a school outbreak would substantially affect the outcome of a newly forming epidemic is in doubt (*20*). In this study, we used stochastic models to estimate the expected size of an outbreak in a school, depending on the delay between detection and implementation of complete school outbreak-response vaccination campaign.

## Methods

We based our stochastic model design on a combined compartmental and individual-based approach (Figure 1). To set our baseline models, we calibrated the model parameters to fit the data from a real school measles outbreak during which no outbreak-response vaccination campaign was implemented. Although detection of an outbreak depends on notification and surveillance policies, we defined the beginning of the outbreak as the day on which the index case-patient showed clear signs of the disease because that is an objective starting point for the outbreak. We also defined the vaccination delay as the interval between the beginning of the outbreak and the day that the vaccination campaign was implemented at the school. For simplicity, we assumed that all nonvaccinated students were vaccinated on the day of implementation of the campaign. We used the calibrated model parameter values (Table) and investigated the outbreak size distribution by varying the vaccination delay. We computed 100,000 simulation runs for each model with different vaccination delays, ranging from day 0 (the same day that the outbreak began) to 150 days, in intervals of 2 days; day 0 meant that the school outbreak-response vaccination campaign was implemented at the beginning of the outbreak.

**Figure 1:** Schematic diagram of stochastic outbreak models to estimate the expected size of a measles outbreak in a school, depending on the delay between detection and implementation of a complete school outbreak-response vaccination campaign. Susceptible persons (susceptibles) become affected if they are infected and become vaccinated after vaccination is implemented. Vaccinated persons (vaccinated) can also be infected but with lower probability than susceptible persons. Those who become affected are followed individually, each with their own transmission and clinical time lines.

**Table:** Definitions of measles outbreak model parameters with assumed values and probability distribution*

Model parameter	Notation	Value/distribution	Reference
Duration of incubation period (from distribution)	D_inc_	Log normal (2.3,0.2); 7–14 d after infection, mode 10 d.	(*21*–*23*)
Duration of latent period (distribution)	D_lat_	(D_inc_ − 4) + normal (0.7); latent period ends ≈4 d before symptom onset.	(*21*–*23*)
Duration of infectious period (from distribution)	D_inf_	(D_inc_ + 4 − D_lat_) + normal (0.7); infectious period ends ≈4 d after symptom onset.	(*21*–*23*)
Duration of symptomatic period (from distribution)	D_symp_	D_lat_ + D_inf_ − Dinc; assumes that symptomatic period ends at same time as infectious period.	(*21*–*23*)
Duration of period to build up immunity after vaccination (from distribution)	D_imm_	13.2 + normal (3.0); approximates measles-specific IgM positivity rates of 2% and 61% after 1 and 2 weeks of vaccination, respectively.	(*24*)
Number of daily contacts per person (from distribution)	n_cont_	20 + NegBin (0.155,2.2).	(*25*)
Infection probability of a susceptible person after contact with an infectious person	P_inf_	0.12 in the R_0_≈16 scenario; 0.2348 in the R_0_≈31 scenario.	This article (Model Calibration)
Vaccination effectiveness	VE_S_	0.9975.	(*5*)
Infection probability of a vaccinated person after contact with an infectious person	P_inf,vac_	P_inf_ (1 − VE_S_ ); 3 × 10^–4^ in the R_0_≈16 scenario; 5.869 × 10^–4^ in the R_0_≈31 scenario.	This article (Vaccination)

*NegBin, negative binomial probability distribution; R_0_≈16, scenario in which basic reproduction number R_0_≈16 is considered; R_0_≈31, scenario in which basic reproduction number R_0_≈31 is considered.

### The Model

We used a compartmental approach to describe the part of the population not yet infected. We considered 2 subgroups—susceptible and vaccinated—to represent the nonvaccinated and vaccinated school populations, respectively, at the beginning of the outbreak. Susceptible persons were assumed to have been completely unexposed (naive) to measles virus and to have a high probability of becoming infected (P_inf_) if they contacted an infectious person. Vaccinated persons were assumed to have acquired protection from measles virus by vaccination and to have a reduced probability of becoming infected (P_inf,vac_>0) to account for an imperfect vaccine.

For susceptible (or eventually a vaccinated) persons who became infected, the model took an individual-based approach and followed each person from the time of infection until the end of the simulation. Persons who had had measles before the outbreak were included as affected but were already immune to infection from the beginning of the simulation. Each affected person had individual transmission and clinical time lines, depending on time since infection, which varied from one person to another.

#### Disease History Time Lines

From the moment a person became infected, and therefore affected, 2 parallel time lines were updated in 1-day steps. The time lines describe the disease history: transmission and clinical (Figure 1).

The transmission time line describes when the affected person became infectious, after a latent period of duration (D_lat_), and when that person ceased to be infectious because the person recovered after the infectious period of duration (D_inf_) had passed. Durations of these periods were generated randomly from their respective probability distributions (Table). After an affected person recovered, that person was unable become infected again because of acquired life-long immunity. The transmission time line describes the events that dictate the dynamics of the epidemic, but in practice these events are difficult to observe.

In contrast, the clinical time line describes events that can actually be observed. This time line indicates the moment of the rash onset, after the incubation period (D_inc_), and the moment when the affected person recovered from the disease, after the symptomatic period (D_symp_) had passed. The values of these periods for each affected person are drawn from the probability distributions indicated in the Table. For simplicity, we assumed that the symptomatic period ended at the same time that the infectious period ended.

Persons who had been infected before the outbreak were considered recovered at the start of our simulations. That is, they were at the end of their disease time lines and did not contribute to the final size of the outbreak.

#### Infection

We assumed that every person in the school mixed with everybody, i.e., homogeneous mixing, and considered daily contact distribution as shown in the Table. Therefore, each day the number of contacts that an infectious affected person had (n_cont_) was drawn from this distribution. From these contacts, the number of newly infected susceptible and vaccinated persons was computed according to the infection probabilities, P_inf_ and P_inf,vac_, respectively.

#### Vaccination

The percentage of vaccinated persons at the beginning of the outbreak was determined by the BVR, and we assumed that vaccination-acquired protection does not wane with time. The high effectiveness of the measles vaccine (*5*,*26*) implies that outbreaks occur mostly among those who are not vaccinated. The intervention strategy consisted of vaccinating all school children without documented measles vaccination; the goal was 100% vaccination coverage. This strategy influences only the susceptible persons by reducing their probability of becoming infected to P_inf,vac_ = (1-VE_S_)P_inf_, where VE_S_ is the vaccine effectiveness value (Table). Susceptible persons are not protected at the moment of vaccination because the immune response to the vaccine takes time to develop. These persons remain susceptible for a period ([D_imm_]; probability distribution is indicated in the Table) after the vaccination day and then become part of the vaccinated group.

#### Isolation

Usually, persons with measles stay home while recovering from the disease. Therefore, we assumed that after disease symptoms developed (after the incubation period D_inc_), infected persons stopped attending school. This absenteeism prevents further contact at school and further spread of the disease, even if the affected person remains infectious at home. For this study, we ignored the possibility of siblings attending the same school.

### Model Calibration

We calibrated our models by using data from a retrospective cohort study conducted at a public day school in Duisburg, Germany, where a large measles outbreak had occurred in 2006 (*19*,*27*). The first cluster in this epidemic had occurred at that school, providing a typical scenario for the early behavior of an outbreak. The outbreak data consisted of a population of 1,250 schoolchildren 10–21 years of age (median 14 years), of which a high proportion (91.3%–94.3%), were vaccinated; 62 students had a history of measles before the outbreak, and a total of 55 cases were confirmed (*5*,*27*). Because no outbreak-response vaccination campaign had been implemented, we calibrated the infection probability (P_inf_) in our models to describe this situation. Assuming a BVR of 91.3%, calculated by Wichmann et al. (*27*), we adjusted the infection probability (P_inf_) in our models so that the size distribution of large outbreaks from our simulations peaked at 55 cases. The calibrated P_inf_ value in combination with our assumed contact distribution (Table) translated into a basic reproduction number of R_0_≈16, which is consistent with reported basic reproduction number estimations for measles (*1*–*4*). The calibrated P_inf_ value in combination with a BVR of 91.3% yielded roughly an effective reproduction number (R_eff_) as follows: R_eff_≈R_0_[1−(BVR/100%)] = 1.4. In theory, if BVR is high enough that R_eff_ is <1, then 1 case generates on average <1 secondary case, leading to herd immunity effects and no large outbreaks (*1*–*4*,*28*).

In a later study, van Boven et al. (*5*) used a Bayesian method to estimate the BVR at 94.3%. Considering this BVR, a higher infection probability (P_inf_) is needed in our models to reproduce the observed outbreak size, which translates to a higher basic reproduction number, R_0_≈31, needed to produce an outbreak in a population with such a high BVR. The conditions in this scenario yield an R_eff_ of ≈1.8, indicating that our simulated outbreaks spread more quickly and had higher attack rates than in the less contagious case of R_0_≈16 and R_ef_≈1.4. To obtain equivalent outbreak sizes, the attack rate (percentage of affected susceptible persons) has to be higher in a population in which BVR is larger because the number of susceptible children is smaller.

Figure 2 shows the final size distribution histograms for R_0_≈16 and R_0_≈31. The typical bimodal distribution predicted by stochastic models appears in both scenarios (*28*) but is most pronounced for the case of R_0_≈31. The bimodal distribution arises because it is always possible that chance events will cause a new outbreak to die out before becoming large. We interpreted the local minimum in both distributions of Figure 2 (i.e., an outbreak size of 20) as a natural but arbitrary limit to distinguish between small and large outbreaks. Therefore, although both distributions show a most probable large outbreak size of 55 persons, in the R_0_≈16 model, 39% of the simulated outbreaks instances become large outbreaks, and in the R_0_≈31 model, 64% of the instances become large.

**Figure 2:** Measles outbreak size histograms calculated with calibrated models. The y-axis indicates the number of model instances counted in their corresponding outbreak size histogram bin, indicated in the x-axis. The dotted line indicates the limit from which large and small outbreaks are defined. BVR, baseline vaccination ratio; R_0_, reproduction number, R_eff_, effective reproduction number.

## Results

Figure 3 shows the final size distribution of outbreaks as a function of the model vaccination delay indicated in the *x*-axis for the R_0_≈16 model. Because of the stochastic nature of the model, as a result of chance events, some outbreaks died out before the intervention was implemented, as could happen in real life. To study the effect of the intervention, we considered only those outbreaks that were still developing at the moment of vaccination. The longer it took to implement the vaccination campaign, the more the outbreak size distributions shifted toward larger outbreaks. Outbreaks were expected to remain small (<20 infected children) if the vaccination delay was 12–24 days. Although the reaction must be quick to avoid an outbreak involving >20 children, a reaction as late as 50 days reduced the final size of large outbreaks in 95% of the simulations. With vaccination delays of >80–90 days, the expected outbreak size was similar to that when no vaccination campaign was implemented.

**Figure 3:** Distribution of measles outbreak sizes as function of vaccination delay for models with basic reproduction number (R_0_) of ≈16 and baseline vaccination ratio (BVR) of 91.3% (effective reproduction number ≈1.4). We considered the outbreaks that were still ongoing at the day of implementation of the outbreak-response vaccination campaign and not those that had spontaneously died out earlier by chance. For every given vaccination delay, the squares indicate the most likely large outbreak size, and the thick solid line indicates the median outbreak size value. The thin solid lines indicate 25th and 75th percentiles, and the tiny dotted lines indicate 5th and 95th percentiles of the outbreak size distribution as a function of vaccination delay. The dashed line shows the outbreak size from the observed data, and the dotted line indicates the chosen limit to separate large and small outbreaks.

We also considered the scenario described by van Boven et al. (*5*), who based the R_0_≈31 estimate on the same school outbreak with a high BVR (94.3%). The high R_0_ value of 31 implies that the infection is more contagious than that in our R_0_≈16 model, leading to a higher effective reproduction number. Figure 4 shows that the outbreak size distributions shifted toward larger outbreaks if the vaccination delay was longer, similar to the R_0_≈16 model. However, because of the higher infectiousness of the disease, outbreaks spread more quickly than in the models with R_0_≈16. This provided only a small time frame of 6–14 days to implement the vaccination campaign if the outbreak size was to be kept small (<20 persons). In 95% of the simulations, a vaccination campaign implemented as late as 40 days after start of the outbreak reduced the final size of the outbreak. There was almost no difference between implementing an outbreak-response campaign after ≈60 days and not implementing one at all.

**Figure 4:** Distribution of measles outbreak sizes as function of vaccination delay for models with basic reproduction number (R_0_) of ≈31 and baseline vaccination ratio (BVR) of 94.3% (effective reproduction number ≈1.8). We considered the outbreaks that were still ongoing at the day of implementation of the outbreak-response vaccination campaign and not those that had spontaneously died out earlier by chance. For every given vaccination delay, the squares indicate the most likely large outbreak size, and the thick solid line indicates the median outbreak size value. The thin solid lines indicate 25th and 75th percentiles, and the tiny dotted lines indicate 5th and 95th percentiles of the outbreak size distribution as a function of vaccination delay. The dashed line shows the outbreak size from the observed data, and the dotted line indicates the chosen limit to separate large and small outbreaks.

From those outbreaks that did not die out before the vaccination campaign was implemented, we computed the percentage of those that would become large. This percentage can be interpreted as the probability that an outbreak will become large. The dependency of this percentage on the vaccination delay is shown in Figure 5, where a range limited by the larger and smaller R_0_ cases is considered. The large outbreak percentage was reduced to 31%–59% if the outbreak-response vaccination campaign was implemented with a delay of 28 days, and it was reduced further if the campaign was implemented earlier: 17%–42% with a 21-day delay, 7%–23% with a 14-day delay, and 1%–7% with a 7-day delay. Although the percentage of large outbreaks became increasingly larger as delay to vaccination became larger, later implementation of outbreak-response vaccination campaigns might still substantially reduce the size of large outbreaks (Figure 3 and Figure 4).

**Figure 5:** Percentage of measles outbreaks that become large for the indicated models. We considered those outbreaks that are ongoing at the moment of implementation of the vaccination campaign, indicated by the vaccination delay in the x-axis. BVR, baseline vaccination ratio; R_0_≈16, scenario in which basic reproduction number R_0_≈16 is considered; R_0_≈31, scenario in which basic reproduction number R_0_≈31 is considered; R_eff_, effective reproduction number.

To extend our study to different settings, we ran simulations in the same school setting and assumed various BVRs, the lowest being 80%, comparable to the current situation in Europe (*8*) (Figure 6). Outbreaks with a higher R_0_ developed more quickly under the same initial conditions and did not become large if the BVR was high enough to achieve herd immunity (>93.8% and >96.8% for R_0_≈16 and R_0_≈31, respectively). For lower BVRs, the final size was larger and outbreaks spread more quickly because of a larger effective reproduction number. Therefore, the time frame to react is smaller; e.g., if BVR = 80% and R_0_≈16, a 1-week delay is already long enough to expect large outbreaks, but a reaction within 3–4 weeks might substantially reduce the outbreak size. Some outbreak sizes were larger than the number of nonvaccinated children in the population, which is explained by imperfect vaccine producing an effect similar to a lower BVR.

**Figure 6:** Measles outbreak size ranges as function of vaccination delay, for models with basic reproduction number R_0_≈16 (A) and R_0_≈31 (B) in the same school setting with various baseline vaccination ratios (BVRs). The ranges shown are between the 95th and 50th percentiles of the outbreak size distribution as a function of vaccination delay and are calculated for each BVR indicated.

We considered a school of 1,250 children, but there are many smaller institutions. In a simulation for a school of 500 children, while conserving the proportions of children with a history of measles and BVR, the boundary separating large and small outbreaks changed to 13 persons. However, results regarding the timing of vaccination remained approximately the same.

## Discussion

We calculated results for 2 reproduction number values, 31 and 16, which belong to a range that is high when compared with published estimates of the basic reproduction number of measles (R_0_≈8–18) (1–4). This comparison indicates that our results are rather conservative with regard to how quickly the intervention should be implemented. However, it must be noted that our results apply to schools in Europe for which BVRs were average (>80%) before the outbreak (*8*).

When we considered institutions with smaller populations than that considered in our study, timing for vaccination remained roughly the same. This finding is explained by the association between the time to react effectively and the generation interval of infection, which is the average time it will take for a newly infected person to infect someone else. The generation interval will depend mostly on the within-host disease development, as long as children have enough contacts to transmit the disease, even in schools with ≈300 children (*25*).

We considered our contact structure to be homogeneous mixing. More intricate contact networks, such as those considering clustering in the different classrooms of the school, popularity of some children, and household contacts with siblings attending the same school, might better resemble reality. Some network structures, contact rates, and superspreading events can influence the speed and growth of an epidemic (*29*). However, our baseline simulations had peak incidence during weeks 6–11 (16th and 84th percentiles, respectively), mode 7 weeks, for the R_0_≈31 scenario; and weeks 6–14, mode 8 weeks, for the R_0_≈16 scenario. These data are consistent with the real outbreak described by Wichmann et al. (*27*), indicating that the rate of disease spread in our simulations was similar to that in the real outbreak.

At the moment of vaccination, a person might already be infected but not yet symptomatic or might become infected soon after vaccination, before immunity has had time to develop. We assume that in these cases the disease progresses in the same way it does in nonvaccinated persons. However, there is evidence that vaccinating during the incubation period might mitigate the symptoms of measles infection (*30*). The vaccine might act as postexposure prophylaxis, potentially reducing the infectiousness of the vaccinated persons.

In many schools in Europe a BVR >80% can be found, but in communities with rather low BVRs (e.g., because of religious or philosophic beliefs), measles virus spreads much quicker. For example, in an outbreak originating in an anthroposophic community in Austria during 2008, with a BVR of 0.6%, of the 123 cases in the anthroposophic school of that community, 96% occurred during the first 4 weeks of the outbreak (*31*), indicating that most cases were either second or third generation (the index case is considered the first generation). An outbreak-response vaccination campaign during such a rapidly spreading outbreak would be effective in a school setting only if implemented within 1 average generation interval (≈9 days) to avoid third-generation infection, which is logistically difficult and requires 100% compliance. However, because school outbreaks are often part of larger community outbreaks, vaccination activities focused on susceptible persons should be promoted in the broader community at any time to prevent further spread of the disease, independent from the time of a school outbreak.

In our study, we assumed 100% compliance to the vaccination strategy. But high compliance to a vaccination campaign cannot be expected in regions with low BVRs associated with religious or philosophical beliefs that are opposed to vaccination. The compliance needed for an intervention to be effective should ensure herd immunity (≈95% vaccinated children). Other complementary measures can be implemented to control measles outbreaks at schools when no vaccination compliance is expected. For example, a measure such as the temporary exclusion of students who lack documented vaccination or whose parents do not agree to vaccination of their children might have a limited effect on preventing further spread of measles by itself (*20*,*32*). However, a combination of timely vaccination of all susceptible students at school who agree to be vaccinated and temporary exclusion of those who do not would be the most effective way to control a measles outbreak in a school.

In conclusion, we computed the possible outcomes of a measles outbreak in a school according to the vaccination delay, assuming that all potentially susceptible children in the school were vaccinated on the same day. We found that it is possible to reduce the number of cases during a measles outbreak in a school by applying a schoolwide vaccination strategy within a realistic time frame. Subsequently, because disease tends to spread in schools during the early stages of a city outbreak, reducing the effects of school outbreaks should help reduce the extent of the outbreak in the larger community. We also showed that BVRs that are high (>80%) but not high enough to achieve herd immunity (≈95%) keep the number of susceptible persons low, reduce the size of an outbreak, and reduce the speed at which the disease spreads, thereby increasing the time frame for mounting an effective intervention.

## References

[1] Anderson RM, May RM. Vaccination and herd immunity to infectious diseases. Nature. 1985;318:323–9. 10.1038/318323a03906406

[2] Anderson RM, May RM. Infectious diseases of humans: dynamics and control. New York: Oxford University Press; 1991. p. 70.

[3] Mossong J, Muller CP. Estimation of the basic reproduction number of measles during an outbreak in a partially vaccinated population. Epidemiol Infect. 2000;124:273–8. 10.1017/S095026889900367210813153PMC2810911

[4] Wallinga J, Lévy-Bruhl D, Gay NJ, Wachmann CH. Estimation of measles reproduction ratios and prospects for elimination of measles by vaccination in some western European countries. Epidemiol Infect. 2001;127:281–95. 10.1017/S095026880100601X11693506PMC2869748

[5] van Boven M, Kretzschmar M, Wallinga J, O’Neill PD, Wichmann O, Hahné S. Estimation of measles vaccine efficacy and critical vaccination coverage in a highly vaccinated population. J R Soc Interface. 2010;7:1537–44. 10.1098/rsif.2010.008620392713PMC2988255

[6] van den Ent MM, Brown DW, Hoekstra EJ, Christie A, Cochi SL. Measles mortality reduction contributes substantially to reduction of all cause mortality among children less than five years of age, 1990–2008. J Infect Dis. 2011;204(Suppl 1):S18–23. 10.1093/infdis/jir08121666160

[7] World Health Organization. Weekly Epidemiological Record. 2002 Feb 15; 77, 7 [cited 2012 Apr 23]. http://www.who.int/wer/2002/wer7707/en/index.html

[8] European Center for Disease Prevention and Control. European monthly measles monitoring, March 2012. Surveillance reports; 2012 Mar 19 [cited 2010 Apr 23]. http://ecdc.europa.eu/en/publications/Publications/Forms/ECDC_DispForm.aspx?ID=843&MasterPage=1

[9] Steffens I, Martin R, Lopalco P. Spotlight on measles 2010: measles elimination in Europe—a new commitment to meet the goal by 2015. Euro Surveill. 2010;15:50. 10.1093/ije/26.3.66221172176

[10] Aylward RB, Clements J, Olivé JM. The impact of immunization control activities on measles outbreaks in middle and low income countries. Int J Epidemiol. 1997;26:662–9. 10.1093/ije/26.3.6629222794

[11] Grenfell BT, Bjørnstad ON, Kappey J. Travelling waves and spatial hierarchies in measles epidemics. Nature. 2001;414:716–23. 10.1038/414716a11742391

[12] Strebel PM, Cochi SL. Waving goodbye to measles. Nature. 2001;414:695–6. 10.1038/414695a11742373

[13] Broutin H, Mantilla-Beniers NB, Simondon F, Aaby P, Grenfell BT, Guégan J-F, Epidemiological impact of vaccination on the dynamics of two childhood diseases in rural Senegal. Microbes Infect. 2005;7:593–9. 10.1016/j.micinf.2004.12.01815820150

[14] Grais RF, DE Radiguès X, Dubray C, Fermon F, Guerin PJ. Exploring the time to intervene with a reactive mass vaccination campaign in measles epidemics. Epidemiol Infect. 2006;134:845–9. 10.1017/S095026880500571616438743PMC2870458

[15] Grais RF, Ferrari MJ, Dubray C, Bjørnstad ON, Grenfell BT, Djibo A, Estimating transmission intensity for a measles epidemic in Niamey, Niger: lessons for intervention. Trans R Soc Trop Med Hyg. 2006;100:867–73. 10.1016/j.trstmh.2005.10.01416540134

[16] Grais RF, Conlan AJK, Ferrari MJ, Djibo A, Le Menach A, Bjørnstad ON, Time is of the essence: exploring a measles outbreak response vaccination in Niamey, Niger. J R Soc Interface. 2008;5:67–74. 10.1098/rsif.2007.103817504737PMC2605500

[17] Alberti KP, King LA, Burny M-E, Ilunga BK, Grais RF. Reactive vaccination as an effective tool for measles outbreak control in measles mortality reduction settings, Democratic Republic of Congo, 2005–2006. In Health. 2010;2:65–8. 10.1016/j.inhe.2009.12.00924037053

[18] World Health Organizaion. Response to measles outbreaks in measles mortality reduction settings. Immunization, Vaccines and Biologicals; 2009 [cited 2012 Apr 23]. http://www.who.int/immunization/documents/diseases/measles_who_ivb_09_03/en/index.html23785742

[19] Wichmann O, Siedler A, Sagebiel D, Hellenbrand W, Santibanez S, Mankertz A, Further efforts needed to achieve measles elimination in Germany: results of an outbreak investigation. Bull World Health Organ. 2009;87:108–15. 10.2471/BLT.07.05018719274362PMC2636188

[20] Bätzing-Feigenbaum J, Pruckner U, Beyer A, Sinn G, Dinter A, Mankertz A, Spotlight on measles 2010: preliminary report of an ongoing measles outbreak in a subpopulation with low vaccination coverage in Berlin, Germany, January–March 2010. Euro Surveill. 2010;15:13:pii:19527.20394713

[21] Richardson M, Elliman D, Maguire H, Simpson J, Nicoll A. Evidence base of incubation periods, periods of infectiousness and exclusion policies for the control of communicable diseases in schools and preschools. Pediatr Infect Dis J. 2001;20:380–91. 10.1097/00006454-200104000-0000411332662

[22] Heymann DL. Control of communicable diseases manual. Washington (DC): American Public Health Association; 2008. p. 349.

[23] LCI Measles, October 2010. In: Steenbergen JE van, Timen A, Beaujean DJMA, editor. LCI-guidelines infectious disease control edition 2011. Bilthoven (the Netherlands): LCI, Coordinator Infectious Disease Netherlands, 2011. P. 382–392.

[24] Helfand RF, Kebede S, Gary HE Jr, Beyene H, Bellini WJ. Timing of development of measles-specific immunoglobulin M and G after primary measles vaccination. Clin Diagn Lab Immunol. 1999;6:178–80.1006665010.1128/cdli.6.2.178-180.1999PMC95683

[25] Mikolajczyk RT, Akmatov MK, Rastin S, Kretzschmar M. Social contacts of school children and the transmission of respiratory-spread pathogens. Epidemiol Infect. 2008;136:813–22. 10.1017/S095026880700918117634160PMC2870867

[26] Uzicanin A, Zimmerman L. Field effectiveness of live attenuated measles-containing vaccines: a review of published literature. J Infect Dis. 2011;204(Suppl 1):S133–48. 10.1093/infdis/jir10221666154

[27] Wichmann O, Hellenbrand W, Sagebiel D, Santibanez S, Ahlemeyer G, Vogt G, Large measles outbreak at a German public school, 2006. Pediatr Infect Dis J. 2007;26:782–6. 10.1097/INF.0b013e318060aca117721371

[28] Bailey NTJ. The total size of a general stochastic epidemic. Biometrika. 1953;40:177–185. doi: http://dx.doi.org/10.1093/biomet/40.1-2.177

[29] Lloyd-Smith JO, Schreiber SJ, Kopp PE, Getz WM. Superspreading and the effect of individual variation on disease emergence. Nature. 2005;438:355–9. 10.1038/nature0415316292310PMC7094981

[30] Sakuta H, Sawada S, Kuroki Y. Severity of measles among patients with incidental postexposure vaccination. Jpn J Infect Dis. 2008;61:304–6.18653975

[31] Schmid D, Holzmann H, Schwarz K, Kasper S, Kuo H-W, Aberle SW, Measles outbreak linked to a minority group in Austria, 2008. Epidemiol Infect. 2010;138:415–25. 10.1017/S095026880999060419678973

[32] Wadl M, Siedler A, Krämer W, Haindl ME, Gebrande S, Krenn-Lanzl I, Measles transmission from an anthroposophic community to the general population, Germany 2008. BMC Public Health. 2011;11:474. 10.1186/1471-2458-11-47421676265PMC3132164

# Research article: Effectiveness and Timing of Vaccination during School Measles Outbreak

**DOI:** 10.3201/eid1809.111578

**Authors:** Axel Antonio Bonačić Marinović, Corien Swaan, Ole Wichmann, Jim van Steenbergen, Mirjam Kretzschmar

**Affiliations:** National Institute for Public Health and the Environment (RIVM), Bilthoven, the Netherlands (A.A. Bonačić Marinović, C. Swaan, J. van Steenbergen, M. Kretzschmar);; University Medical Centre Utrecht, Utrecht, the Netherlands (A.A. Bonačić Marinović, M. Kretzschmar);; Robert Koch Institute, Berlin, Germany (O. Wichmann);; and Leiden University Medical Centre, Leiden, the Netherlands (J. van Steenbergen)

## Medscape CME Quiz

## Earning CME Credit

To obtain credit, you should first read the journal article. After reading the article, you should be able to answer the following, related, multiple-choice questions. To complete the questions (with a minimum 70% passing score) and earn continuing medical education (CME) credit, please go to www.medscape.org/journal/eid. Credit cannot be obtained for tests completed on paper, although you may use the worksheet below to keep a record of your answers. You must be a registered user on Medscape.org. If you are not registered on Medscape.org, please click on the New Users: Free Registration link on the left hand side of the website to register. Only one answer is correct for each question. Once you successfully answer all post-test questions you will be able to view and/or print your certificate. For questions regarding the content of this activity, contact the accredited provider, CME@medscape.net. For technical assistance, contact CME@webmd.net. American Medical Association’s Physician’s Recognition Award (AMA PRA) credits are accepted in the US as evidence of participation in CME activities. For further information on this award, please refer to http://www.ama-assn.org/ama/pub/category/2922.html. The AMA has determined that physicians not licensed in the US who participate in this CME activity are eligible for *AMA PRA Category 1 Credits*™. Through agreements that the AMA has made with agencies in some countries, AMA PRA credit may be acceptable as evidence of participation in CME activities. If you are not licensed in the US, please complete the questions online, print the certificate and present it to your national medical association for review.

## Article Title: Effectiveness and Timing of Vaccination during School Measles Outbreak

## CME Questions

**1. You are on the health advisory board of your local elementary school district. Two students in one fourth-grade class were diagnosed with measles today. The overall rate of vaccination against measles in the school is 90%.**

**What should you consider regarding vaccination and infection with measles?**

A. The estimated basic reproduction number R_0_ for measles is in the range of 12 to 40

B. The case-fatality rate in industrialized countries is approximately 10%

C. Vaccination rates in Europe generally exceed 95%

D. Vaccinating during the incubation period of measles has no salutary effect

**2. You estimate that the R_0_ for measles is actually 16. Based on the results of the current study, what is the maximum amount of time that can pass before an outbreak response vaccination (ORV) is initiated in order to limit measles infection to no more than 20 children?**

A. 2–4 days

B. 6–10 days

C. 11–15 days

D. 12–24 days

**3. Based on the results of the current study, what is the maximum amount of time that can pass before an ORV is initiated in order to affect the size of the measles outbreak at all?**

A. 25 days

B. 35 days

C. 50 days

D. 65 days

**4. Based on the results of the current study, when can you expect the peak incidence of measles if an outbreak develops?**

A. 1 to 3 weeks

B. 4 to 7 weeks

C. 6 to 14 weeks

D. 20 to 25 weeks

## Activity Evaluation

**Table:** 

**1. The activity supported the learning objectives.**				
Strongly Disagree				Strongly Agree
1	2	3	4	5
**2. The material was organized clearly for learning to occur.**				
Strongly Disagree				Strongly Agree
1	2	3	4	5
**3. The content learned from this activity will impact my practice.**				
Strongly Disagree				Strongly Agree
1	2	3	4	5
**4. The activity was presented objectively and free of commercial bias.**				
Strongly Disagree				Strongly Agree
1	2	3	4	5
